# Notes from Dental Practice

**Published:** 1869-06

**Authors:** 


### ARTICLE V.

### *Notes from Dental Practice.*

*Disease of the Antrum.* History and Nature of Case.—Patient a resident of North Carolina, aged 38 years, of a nervous temperament, and, though inclined to be fleshy, possessing an impaired constitution. He gives the following history of his case: "About the middle of July last, I became overheated during the day and the weather being extremely warm at night, I threw a quilt upon the floor of the piazza and fell asleep having no other clothes on except a shirt and pants, which were wet with perspiration. Upon awakening I found myself almost frozen, and the next day was afflicted with a heavy, dull headache, and all the symptoms of a severe cold. At the end of two weeks the discharge from the nose ceased, but this cessation was followed by a fever, periodical in its character, which continued for a week when the pain in my head increased to such a degree as to almost cause delirium.

My family physician was called in and relief was obtained in a day or two, the pain in my head becoming much less severe, but the fever continued for a month longer resisting all remedies which were administered to check it.

I had for some time suffered from bad teeth and old roots, one of which was extracted, and for this reason my physician requested me to apply to the dentist, which I did. Dr. T., the dentist, upon examining my mouth pronounced my disease to be suppuration of the lining membrane of the antrum. He extracted all of the diseased teeth and roots, and made a hole through the bone of the jaw into the antrum, using for this purpose the cavity of the third tooth back of the eye-tooth; through this opening a good deal of water mixed with blood escaped. The Doctor then syringed the cavity through this opening twice a day with water, washing matter out of the nostril of the affected side, which gave me some relief, after which he injected rose water. At the end of a week from the time the water was first injected, recourse was had to sulphate of zinc, 6 grs. to the ounce of water, which was thrown into the cavity once a day.

As this preparation did not appear to have the desired effect, an application of nitrate of silver 1 gr. to the ounce of water, was injected, but after using one ounce of this preparation, it caused me so much pain that I discontinued it. Something has of late made it appearance in my nose, obstructing the free passage, which on being removed was pronounced by Dr. T. to be "polypus," but as it has not returned may have been thick mucous matter. On syringing the antrum there appears to be at times some little flocculent substance blown about by the injection near the hole which leads from the antrum into the nostril. I am at times better, then worse, and my last attack has been the most severe; for six weeks past I have been confined to the bed for more than half the time, but at present I can walk about for some hours every morning.

My forehead and eyes are somewhat swollen, my right eye almost shut; this eye, before my last attack had not been affected, as the disease was confined to the left side of my head and the left eye. I have been subject to pains in the diseased left eye for a good many years, called "sun-pain," which quinine would generally relieve, and I have used quinine this time with benefit. My hands and feet are cold when I am in much pain, but as soon as I get into a perspiration I experience relief. When syringing the cavity, if I do not place the end of the nozzle of the syringe in the direction of the opening of the cavity into the nose, I can get but little if any matter out. It was one month from the time I was first taken before the discharge of matter commenced about the root of one of the teeth I had extracted, which was soon followed by the discharge from the nostril of the same side; it now runs into my mouth from the opening made into the antrum, and when it is very thin I cannot prevent its running down my throat and causing great nausea.

It has never discharged much from the nostril of the side first said to be diseased, since the operation upon that side. On the sixth of November I was seized with a chill which was followed by fever, and then the right nostril discharged bloody matter; since that time the right nostril has been constantly discharging, and the left one none of any consequence."

*Treatment.*--From the above description of this case, and without having seen the patient, we conclude that the affection of which he is suffering is either scrofulous, syphilitic or cancerous. The discharge being now from the right nostril is evidence that the bones of the nose are diseased, and the swelling and pain in the eyes and forehead that the disease has invaded the frontal sinuses.

The following treatment is thereforere commended: Injection into the antrum of either Lugol's solution or the permanganate of potash; inhalation of iodine once or twice a day; tincture of iodine externally applied; and of the syrup of the iodide of iron gtt. x, iodide of potass. gr.viij, cod liver oil a dessert spoonful, this prescription to be taken three times a day. In addition to the above, benefit may also be derived from some bitter tonic, such as tincture of cinchonæ or tincture of gentian, half an hour before each meal.

